# Supplementary material for: From sequence to enzyme mechanism using multi-label machine learning
Source: BMC Bioinformatics. 2014 May 19;15:150. doi: 10.1186/1471-2105-15-150 (PMC4229970; doi:10.1186/1471-2105-15-150)
Supplement: Additional file 2 — Java code of ml2db. Additional file ml2db_code.tar.gz contains the Java source code to run the multi-label machine learning experiments and save the results to database. The code’s Javadoc is included. [file 1471-2105-15-150-S2.zip › additional file 2/ml2db/ml2db/doc/main/ml2db/al/learning/package-use.html]

Uses of Package main.ml2db.al.learning


JavaScript is disabled on your browser.


- Overview
- Package
- Class
- Use
- Tree
- Deprecated
- Index
- Help

- Prev
- Next

- Frames
- No Frames

- All Classes

# Uses of Package main.ml2db.al.learning

- Packages that use main.ml2db.al.learning

  | Package | Description |
  | --- | --- |
  |  |  |
  | --- | --- |
  | main.ml2db.al |  |
  | main.ml2db.al.learning |  |
  | test.ml2db.al |  |
  | test.ml2db.al.utility.confidence |  |
  | test.ml2db.al.utility.globaldistance |  |
  | test.ml2db.al.utility.neighbourhood |  |
  | test.ml2db.al.utility.pickers |  |
- Classes in main.ml2db.al.learning used by main.ml2db.al

  Class and Description

  ExperimentDetails

  Manages the data for an active learning experiment: experiment id, arff file
  used, algorithm for training/testing, type of distance used (Euclidean etc.),
  type of utility metric used to pick the next query instance to be labelled,
  timestamp etc.
- Classes in main.ml2db.al.learning used by main.ml2db.al.learning

  Class and Description

  AlExperimenter

  Class

  AlOutputManager

  Class
- Classes in main.ml2db.al.learning used by test.ml2db.al

  Class and Description

  AlExperimenter

  Class

  ExperimentDetails

  Manages the data for an active learning experiment: experiment id, arff file
  used, algorithm for training/testing, type of distance used (Euclidean etc.),
  type of utility metric used to pick the next query instance to be labelled,
  timestamp etc.
- Classes in main.ml2db.al.learning used by test.ml2db.al.utility.confidence

  Class and Description

  ExperimentDetails

  Manages the data for an active learning experiment: experiment id, arff file
  used, algorithm for training/testing, type of distance used (Euclidean etc.),
  type of utility metric used to pick the next query instance to be labelled,
  timestamp etc.
- Classes in main.ml2db.al.learning used by test.ml2db.al.utility.globaldistance

  Class and Description

  ExperimentDetails

  Manages the data for an active learning experiment: experiment id, arff file
  used, algorithm for training/testing, type of distance used (Euclidean etc.),
  type of utility metric used to pick the next query instance to be labelled,
  timestamp etc.
- Classes in main.ml2db.al.learning used by test.ml2db.al.utility.neighbourhood

  Class and Description

  ExperimentDetails

  Manages the data for an active learning experiment: experiment id, arff file
  used, algorithm for training/testing, type of distance used (Euclidean etc.),
  type of utility metric used to pick the next query instance to be labelled,
  timestamp etc.
- Classes in main.ml2db.al.learning used by test.ml2db.al.utility.pickers

  Class and Description

  ExperimentDetails

  Manages the data for an active learning experiment: experiment id, arff file
  used, algorithm for training/testing, type of distance used (Euclidean etc.),
  type of utility metric used to pick the next query instance to be labelled,
  timestamp etc.

- Overview
- Package
- Class
- Use
- Tree
- Deprecated
- Index
- Help

- Prev
- Next

- Frames
- No Frames

- All Classes
